# The A. D. A. Meeting for 1887

**Published:** 1887-07

**Authors:** 


### THE A. D. A. MEETING FOR 1887.

Many difficulties lay in the way of a satisfactory solution of the question of the Association meeting for this year, and there was a wide diversity of opinion as to what is best to be done. The embarrassment chiefly arose from the fact that the majority of those prominent in the councils of the Association are deeply interested in the success of the International Medical Congress, which meets in Washington early in September. It was feared that both could not be made a success, and that one meeting must be held at the expense of the other. Probably a majority of the members would have favored passing the meeting for the year. There were those, however, who believed that this would be prejudicial to the future of the Association, and would be an exhibition of weakness unworthy a society which stands as the representative of American dentistry, both at home and abroad. But whatever the expediency of the occasion, an examination of the organic law of the Association was sufficient to convince any one that the meeting could not legally be passed. The Executive Committee could not do otherwise than to call it, although the time and place, within certain limits, were in case of emergency left to their discretion.

We think the matter has been wisely decided. Niagara was the birth-place of the Association, and its meetings have been more frequently held there than in any other place. It was not best to go south this year, because of distracting interests, for when we do go to Asheville, or to any southern city, we wish to have nothing in the way that shall render the issue of the meeting problematical. As to the time, perhaps that most favorable would have been the week immediately preceding the meeting of the Congress, were it not that the Southern Society had already pre-empted that date. To call the meeting for the week after the Congress would be folly, because all will be exhausted at that time, and because many will then be engaged in the entertainment of foreign and other visitors.

Both time and place are now fixed, and neither is any longer a debatable question. All could not be pleased. The preferences of some must be sacrificed, but we trust that they will accept the decision with cheerfulness. He is not a good member, or possessed of the proper spirit, who will sulk in his tent because he cannot have his own way. All society and all societies exist only by a series of compromises. Individual predilections must be sacrificed to the good of the whole. There has been in the past too much of the rule or ruin disposition, and the Association has suffered by it. This year let us all lay aside every selfish consideration and act in harmony and with unanimity. All cannot probably attend the meeting, but every one who is loyal to the society, and who loves his profession, will do what he can to promote the success of the annual meeting for 1887.

A failure has been predicted by some croakers. Why need this be anticipated ? The same thing was presaged for the meeting of the American Medical Association for this year. That society is far more intimately connected with the Congress than is the A. D. A., for it has assumed control of it and made itself directly responsible for it. The most of its members are at work very hard for the success of the Washington meeting, and every energy is absorbed in preparation for that event. But the meeting of the American Medical Association, held in Chicago, in June, was the most successful that society has ever known. More than fifteen hundred delegates were in attendance, and the number of papers presented was nearly double that of last year's meeting. Why should it not be the same with our own convocation ? It will be the best preparation possible for the September meeting, and the delegates will not go direct to Washington tired and jaded, but will have sufficient time for rest and recuperation.

Our most successful meetings are not usually the largest. The Association meets, not to have a good time, but to advance our professional interests. To this end, one really valuable original paper is worth more than the attendance of a hundred hangers-on. A scientific and instructive debate is not one in which a great number of persons take part, merely that they may have their names recorded in the transactions, but it is the comparison of views by those who are instructed upon the subject. If this year's meeting will witness the presentation of a few valuable papers, and their discussion by men who really have something to say, it will be worth while to attend it. If we can, this year, be freed from the crude, undigested thoughts, the unripened fruit of undisciplined minds, and in their place get the results of a ripe experience, if those who desire to take part in the meeting will make some preparation beforehand and be ready with carefully digested and definite conclusions, if we can for one year be relieved from the infernal constitution tinkering and the by-law debating of those who are capable of nothing higher or better, then we may have a meeting that shall long be memorable in the history of the Association; one that we shall look upon with pride as having advanced the status of our beloved profession and been a blessing to all those who did not, as well as in a greater measure to those who did, attend it. Brethren, shall this be done ?

But it must be understood that there shall be no "bosses" to dominate the sessions. We have had sufficient of that in the past, and now we want a new departure. No one need kindly undertake the task of deciding who shall be the officers for 1888. The Association is quite capable of transacting that business for itself, and he who undertakes the role of dictator will surely come to grief.

The local committee of arrangements have engaged the same rooms that were occupied at the meeting last year. They have done more. There was complaint at the last meeting that some dealers were unable to secure space, others having monopolized everything. There was also annoyance from the fact that the Association was hemmed in by dealers and manufacturers, and was unable to control the passages and door-ways that led to the hall. The local committee has therefore now engaged the whole building, and the Association will itself assign space to the depots. This has not been done as a speculation, or for the purpose of oppressing any, but that the society may be able to control its own hall. Dental dealers who desire accommodations may address the chairman of the local committee of arrangements, Dr. S. A. Freeman, 14 Court St., Buffalo, N. Y.

The hotel arrangements are about the same as last year. The International will be headquarters, and will give special rates of $3.00 per day. The Cataract House will make no deductions. Some of the other hotels will make special rates, which will be announced in the circular to be issued by the Executive Committee. Rooms can be engaged by addressing the chairman of the local committee, as above.

THE LATE ACTION OF THE AMERICAN MEDICAL ASSOCIATION.

In the department of "Current News" will be found the resolutions upon the subject of the medical status of educated dentists, adopted at the late meeting of the American Medical Association, with the remarks of Dr. N. S. Davis, the President of the International Medical Congress, who offered them for consideration. We hope that every intelligent dentist will very carefully study them, entirely master their every detail and fully comprehend their tendency and bearing. If they were spontaneously offered, carefully considered and understandingly adopted in the American Medical Association, they mark a most important era in dental history, and their influence upon our future cannot well be overestimated. If they were approved without due consideration, if they were offered at the instigation of any interested man or class of men, if they were accepted simply because one high in medical councils consented to advocate them and were voted upon in mere routine, they are worse than useless—they are a mockery and a delusion. Hence it behooves us carefully to consider what influences were potent in bringing about this action; whether, in fact, it marks a real change in the medical sentiment, or whether the whole affair is mere claptrap.

We have no positive knowledge of the train of events which brought about this action. The names of influential dentists have been mentioned as having instigated the movement. If this be so, and if it be true that it was in deference to their wishes that these resolutions were passed, we must say that, praiseworthy as doubtless were their motives, it would have been better that they had not put forth their hands to steady the ark of our progress. Unless the resolutions mean an honest expression of thoughtful and enlightened opinion, they are misleading and will tend to errors. We say, therefore, that they should be carefully considered by dentists, and that we must proceed thoughtfully.

Dentistry was digged from a slimy pit. In its early days it was almost entirely mechanical and smacked strongly of charlatanism, as the resolutions intimate. But Harris and Brown and Bond and others, saw the possibilities of a reputable and scientific practice. They believed the future to be pregnant with great things for dentistry, if once its practitioners could be brought to a higher plane—if the crude methods of the day could be crystallized into a consistent system to be followed by educated and intelligent men. They applied to certain medical colleges to commence the teaching of a dental practice founded on medical knowledge, and running in technical, scientific channels. What wonder that they were refused. Was the dentistry of that day a fit associate for scientific medicine? There was no theory of practice whatever. Dentists generally were ignorant, untrained, peripatetic tinkers. Exceptions there were, of course, but the great body of the dentists of that day were devoid of technical knowledge. Medicine was right in refusing to acknowledge the unknown foundling.

The only course left was pursued, and separate colleges were founded and a new, unheard-of degree instituted. In these schools the teaching was at first mainly practical; and not scientific. But we have constantly grown, and the prescience of Harris and his compeers has been vindicated. Educated men came into dentistry, and its practitioners began to be known in the world of science. Our schools became broader in their teachings, and thoughtful medical men began to see what were the possibilities hidden under the at first so unpromising exterior. Some of the great universities recognized the scientific aspect of the new specialty, and saw its near relation to medicine and the liberal professions. Harvard threw open its doors, devised a new degree more consistent with literary training, and now for the first time dentistry took her place in the halls of her mother, medicine. Other great universities followed—Michigan and Pennsylvania—and now a liberal dentistry is a recognized part of true medical teaching.

In England a movement was in the meantime active, which resulted in the placing of dental practice under the direction of medicine. We have not here the space to review the relations of the two in that country, but it must suffice to say that medicine there occupies the position of keeper, or guardian, or warden of dentistry, rather than that of mother, or elder sister. Yet the true nature of an intelligent dental practice was so far recognized in England that when the Congress of 1881 was called, dentistry was assigned a position, although no distinctive dental degree was recognized, and the success of the section so organized, the high scientific character of the papers and discussions within it was so pronounced as materially to advance our status in the eyes of the whole scientific world. The influence of that meeting extended to America, and the American Medical Association established a new section to be devoted exclusively to dentistry, thus practically recognizing legitimate dental practice as an integral portion of medicine.

There was another factor which has been potent in bringing about this changed condition, and that was the legislation which has been secured in many of the States, regulating and restricting dental practice. We are not at all certain that this was not the most powerful influence that had been exerted, for we were now recognized by the sister professions of law, and occupied a definitely established position; we had risen in popular estimation and were respected of the people, without which general acknowledgment all legislation was a mockery, and this has been brought about by the State laws regulating dentistry.

When the Congress was called for 1887, a dental section was formed by the men who were then foremost in its councils, and after the revolution in which the American Medical Association assumed direction, the section was re-established, consolidated and given broader scope. For the past year and a half no department of that great organization has received more deferential treatment, nor had its suggestions and wishes more promptly conceded than has that of the Section of Dental and Oral Surgery. We are free to admit that, personally, we had not at first entire confidence in the sincerity of the profession of amity and good-will. We feared there was more of expediency than ingenuousness in all the professions of confidence and trust and recognition. But time has removed these early impressions, and we are now fully convinced that the members of the American Medical Association and the principals in the Congress have learned to respect the good that is in us, and to extenuate the evil which is constantly growing less, and hence that the late expression of the American Medical Association is an indication of the changed condition of the estimation in which we are henceforth to be held by the great body of medical men.

It is true that there is a faction in medicine headed and voiced by *The Medical Record*, of New York, which, perhaps puffed up by an undue and exaggerated sense of their own importance, can see no good outside their own narrow circle. That journal never misses an opportunity to insult and belittle dentistry, or to throw ridicule upon the claims of its best exemplars. But *The Record* is a bitter opponent of the Congress, and belongs to the prejudiced and bigoted Bourbon wing of medicine which learns nothing, makes no advance, because it is incapable of appreciating its own intolerance or comprehending the progress made by others. Happily *The Record* voices a faction which is rapidly growing less, both in numbers and influence.

We are prepared, then, to accept the resolutions of the American Medical Association as having been offered and unanimously passed in good faith. And now what is their signification? It will be seen by a careful perusal that, for the first time, the diplomas of a dental college are recognized. During all the changes and growths of the past, it has been only those dentists who possessed the medical degree who were admitted to medical equality. It is a step of which few recognize the importance, when another diploma than that of medicine is admitted, even in part, as qualifying for a place in medical practice. This action distinctly recognizes dentistry as a part of medicine, and properly organized dental colleges as teaching a specialty in general medicine, and that is a wonderfully long step in advance, if it be understandingly taken. We have never been of those who clamored for "recognition." We do not wish to be "recognized" for what we are not. But if this be an indication of the estimation in which we are honestly held, we shall greatly rejoice, because it is proof positive of a healthy scientific growth.

But it will be noticed that the American Medical Association proposes to recognize only the graduates of such dental schools as require a preliminary examination for the evidences of a fair degree of literary requirements, and which demand an attendance throughout a first-class college curriculum, with three years of study. These conditions will cover the diplomas of but a portion of our colleges. There are even now some schools which make a pretence of complying with the demands of the National Association of Dental Faculties, but which nullify them in practice. There has been in the past villainous work done by dental schools which pretend to respectability, and which have connected with them men who stand high in the councils of the profession. These schools, pretending to regularity, have graduated foreigners who were utterly ignorant of our language. They have brought our diplomas into disrepute abroad, and made American dentistry a by-word and reproach in foreign countries. Men claiming to be honorable men have practically sold diplomas for a few dirty dollars. They are becoming known, and it is time that their vile practices were yet more fully exposed, and they held up to the reprobation which they deserve. Now is the time for dentistry to put itself in line with real progress; to rid itself of the barnacles which cling to it for mere pelf or power, and to make every school what it should be. If any college will not stand fully abreast of the position outlined by these resolutions, let it be Anathema Maranatha, and its name no more be heard among honorable men. Let us cease taking young men directly from the plough or from the servants' hall, and without scientific training attempt to make professional men of them. Let us accept the liberal conditions offered, and live up to them in good faith. "Noblesse oblige," and if we do not appreciate the present opportunity we shall be unworthy any "recognition" whatever, and should henceforth be content with a position among the hewers of wood and the drawers of water.

